# Exploratory laparoscopy combined with pathological examination in the diagnosis of obscure gastrointestinal bleeding in a child: a case report

**DOI:** 10.1186/s12887-018-1339-9

**Published:** 2018-11-27

**Authors:** Jiande Chen, Bin Zhang, Zhilong Yan, Huaying Zhao, Kaihua Yang, Yong Yin, Lirong Jiang

**Affiliations:** 1 Department of Respiratory Medicine, Shanghai Children’s Medical Center Affiliated to Shanghai Jiao Tong University School of Medicine, No.1678 Dongfang Road, Pudong, 200127 Shanghai China; 2 Department of Gastroenterology, Shanghai Children’s Medical Center Affiliated to Shanghai Jiao Tong University School of Medicine, No.1678 Dongfang Road, Pudong, 200127 Shanghai China; 3 Department of General Surgery, Shanghai Children’s Medical Center Affiliated to Shanghai Jiao Tong University School of Medicine, No.1678 Dongfang Road, Pudong, 200127 Shanghai China

**Keywords:** Iron-deficiency anemia, Melena, Vascular malformations

## Abstract

**Background:**

The diagnosis of obscure gastrointestinal bleeding (OGIB) which is defined as bleeding of unknown origin of the small bowel by routine evaluation in childhood is a challenge.

**Case presentation:**

Here we report a one-year-old Chinese girl who was suspected with idiopathic pulmonary haemosiderosis (IPH) and referred to our department for further diagnosis. Finally she was diagnosed with vascular malformations (VM) by exploratory laparoscopy combined with pathological examination.

**Conclusions:**

Children OGIB could be easily misdiagnosed in the beginning, and OGIB children with active ongoing bleeding may benefit from proceeding directly to exploratory laparoscopy, followed by pathological confirmation of the diagnosis.

## Background

Obscure gastrointestinal bleeding (OGIB) is defined as bleeding of unknown origin that persists or recurs after bidirectional endoscopy and radiologic evaluation of the small bowel [1]. It could be categorized into obscure overt and obscure occult bleeding based on the presence or absence of clinically evident bleeding [2]. Causes of OGIB may potentially include lesions that are overlooked in the esophagus, stomach, and colon during initial workup or lesions in the small intestine that are difficult to visualize with conventional endoscopy and radiologic imaging [1]. After negative endoscopy and colonoscopy, performing small bowel endoscopic investigation by capsule endoscopy (CE) and balloon-assisted enteroscopy (BAE) has a very good diagnostic yield [3]. Intraoperative enteroscopy is currently reserved as a last option, for when other measures cannot identify a bleeding source in selected patients.

This paper presents an unusual case study of a one-year-old girl who presented with OGIB, the subsequent diagnostic challenges encountered and how these were addressed.

## Case presentation

**Fig. 1:**
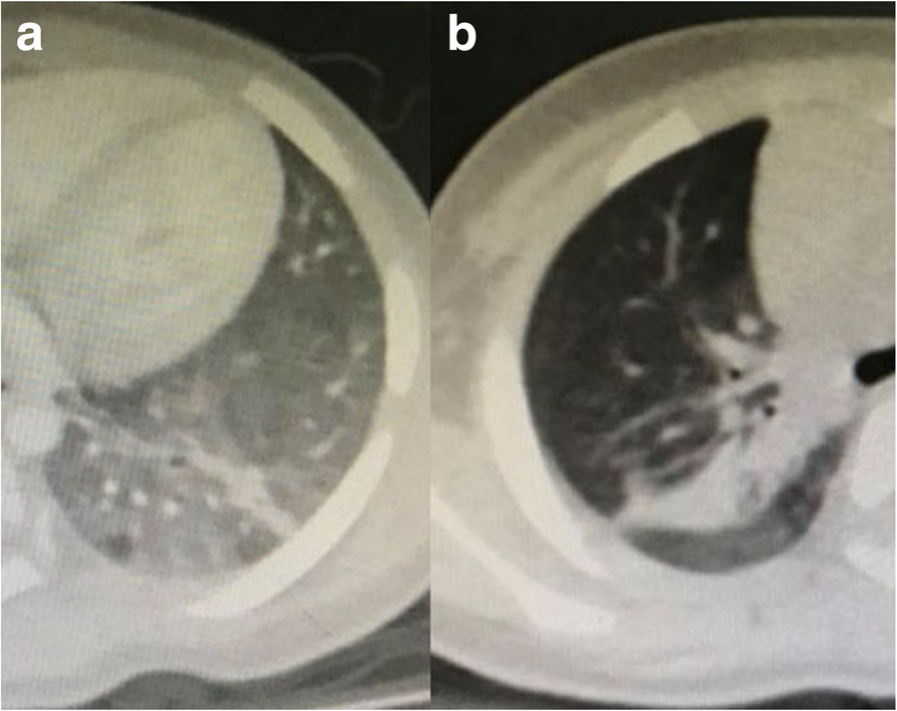
Chest CT. Increased patch density in the left lower lobe (**a**) and the right upper lobe (**b**) of the lung

A nine-month-old Chinese girl presented with one-week history of pallor at a referral hospital where she received a red blood cell transfusion for severe anemia (Hb 3.4 g/dL) and started to treat for iron-deficiency anemia (IDA) after microcytosis (mean corpuscular volume 74.6 fl), hypochromia (mean cell Hb 21.5 pg), and low serum iron concentration (1.28umol/L) were confirmed. On discharge after 1 week of treatment, anemia was corrected (Hb 12.4 g/dL). However, recurrent anemia was observed over a six-month period, even another red blood cell transfusion was given in this period. Positive fecal occult blood test results were intermittent. A chest computed tomography (CT) scan showed the increase of patch density in the left lower lobe (Fig. 1a) and right upper lobe (Fig. 1b) of the lung. Although she had no history of repetitive haemoptysis, chronic cough and dyspnoea, idiopathic pulmonary haemosiderosis (IPH) was entertained and the IDA therapy was discontinued.

Patient was referred to our hospital for further management. Flexible bronchoscopy was performed, but bronchoalveolar lavage examination of blood-stained fluid and hemosiderin-laden macrophages from involved areas was negative. Review of the chest CT scan showed no extensive ground glass opacities and reticular shadows. Therefore, diffuse alveolar haemorrhage was ruled out. Review of the patient’s history found an episode of intermittent melena 1 month after the IDA treatment, and that was considered to be the side effect of the drug by the outpatient doctor. No related family genetic history. Physical exam demonstrated a girl of normal appearance consistent with her ethnicity except pallor. The diagnostic approach for gastrointestinal bleeding was started. However, the patient underwent both upper and lower endoscopy with negative findings in all of the endoscopic examinations. Plain and enhanced CT of abdomen and the technetium-99 m–labeled red blood cell scans were performed. Again, they were all negative. Her symptoms persisted and one red blood cell transfusion was needed each week.

**Fig. 2:**
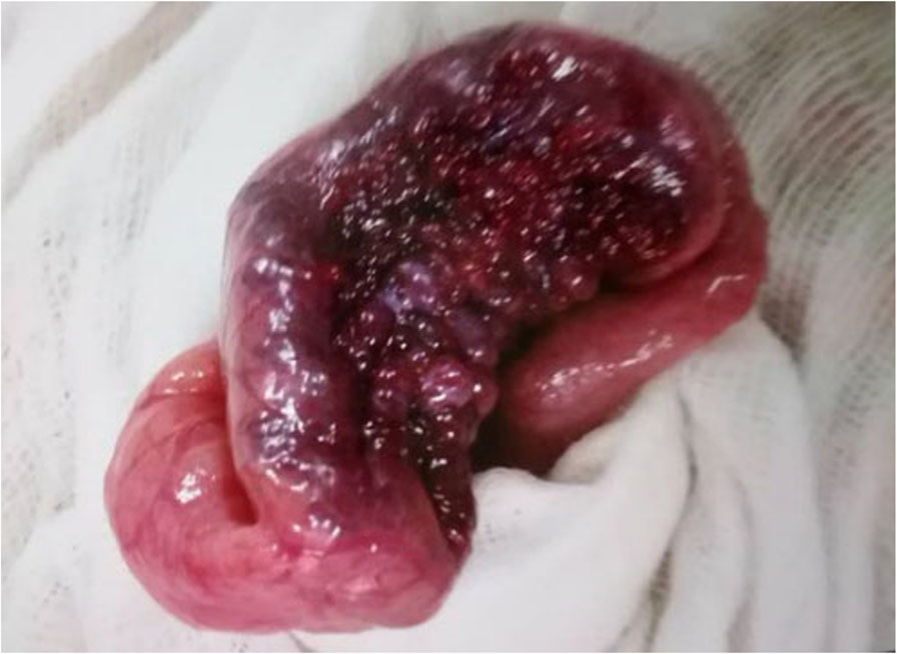
Lesion within the wall of jejunum. A 3 cm lesion with dense blistered protrusions on the surface within the wall of jejunum

**Fig. 3:**
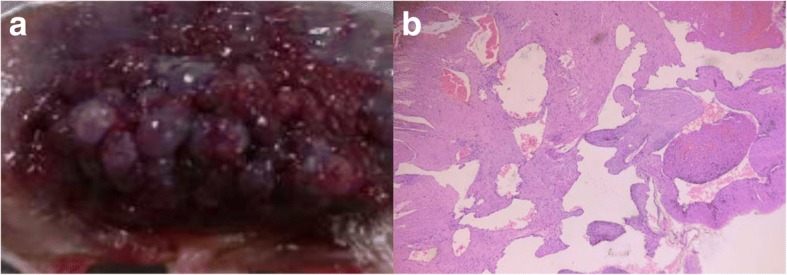
Gross view and microscopic features of intestinal wall VM. Macroscopically, there was a lesion with dense blistered protrusions on the surface within the intestinal wall (**a**). On microscopy, abnormal collections of dilated vascular structures of variable sizes were observed in the lesion (**b**, original magnification × 10)

The department of general surgery was involved in the management and a decision to do surgical exploration with laparoscopy was taken. A 3 cm lesion with dense blistered protrusions on the surface was found within the wall of jejunum (Fig. 2), acting as a lead point, so a jejunal segment was resected and an end to end jejunojejunostomy was performed. Pathological examination indicated a vascular malformations (VM) (Fig. 3). Postoperative period was uneventful and she was discharged home with no complications. There was no recurrence during follow-ups.

## Discussion and conclusions

OGIB from VM in this case affected the delay in diagnosis because of its rarity and limitations in the diagnostic approach in pediatric patients.

Massive gastrointestinal haemorrhage in a child due to VM of the jejunum is very uncommon [4]. To our knowledge this is the second case of an acute gastrointestinal haemorrhage in a child due to VM of the jejunum. Most VM cases may lay a false trail for the clinician because of accompanied IDA with no gastrointestinal symptoms at the initial time [5]. In our case, IDA combined with asymptomatic pulmonary infection misled the diagnosis as IPH. The clinical conditions of our case are reported for the first time.

Syndromes such as the Klippel-Trenaunay syndrome and the blue rubber blebnevus syndrome usually encompass VM as a skin manifestation, so the possibility of visceral lesion may be suspected. In this case, however, the malformations were a unique manifestation without any associated syndrome, which increased the difficulty of diagnosis.

Angiography may detect OGIB lesions and also offers a therapeutic option with embolization if a bleeding lesion is identified. In OGIB patients, the bleeding rate may be slow or intermittent, thereby not allowing identification by either angiography or bleeding scan [6]. A small case series also suggests that the overall yield of provocative angiography is low [7].

CE is currently the preferred test for the initial investigation in patients with OGIB due to its high diagnostic yield [1]. However, this technology requires precision instruments and skilled endoscopic images interpreters. In addition, the increase of the cost-effectiveness, imprecise localization, the risk of capsule retention and a lack of therapeutic capability also restrict the wide application of CE among children patients, particularly in acute cases.

OGIB was a common indication for small bowel endoscopy. The development of BAE represents a decisive breakthrough in the diagnosis and management of small bowel diseases. The overall diagnostic yield of BAE was about 70% [8, 9]. The approach of CE followed by BAE might show a diagnostic yield over 90% [10]. However, this technology has not been widely used in children’s hospitals for concerns regarding safety, design of instruments, training, availability, and a lack of knowledge about its use and relative indications.

The safety and effectiveness of using laparoscopy as the diagnostic and therapeutic tool for OGIB in children have been well established by pediatric literature [11–14]. In the cases of difficult-to-manage or acute bleeding, we may directly resort to laparoscopy for difficult-to-access lesions. Pathological examination should be performed to make a definite diagnosis after lesions resection.

Our experience of successful management of this case suggested that children OGIB combined with asymptomatic pulmonary infection could be easily misdiagnosed as IPH in the beginning, and OGIB children with active ongoing bleeding may benefit from proceeding directly to exploratory laparoscopy, followed by pathological confirmation of the diagnosis.

## Abbreviations

BAE: Balloon-assisted enteroscopy
CE: Capsule endoscopy
CT: Computed tomography
IDA: Iron-deficiency anemia
IPH: Idiopathic pulmonary haemosiderosis
OGIB: Obscure gastrointestinal bleeding
VM: Vascular malformations
